# Augmentation of the Female Reproductive System Using Honey: A Mini Systematic Review

**DOI:** 10.3390/molecules26030649

**Published:** 2021-01-27

**Authors:** Nur Hilwani Ismail, Siti Fatimah Ibrahim, Farah Hanan Fathihah Jaffar, Mohd Helmy Mokhtar, Kok Yong Chin, Khairul Osman

**Affiliations:** 1 School of Biological Sciences, Faculty of Applied Sciences, Universiti Teknologi MARA, Shah Alam 40450, Malaysia; hilwani@uitm.edu.my; 2 Department of Physiology, Faculty of Medicine, Universiti Kebangsaan Malaysia Medical Center, Kuala Lumpur 56000, Malaysia; timi@ukm.edu.my (S.F.I.); farahhanan@ukm.edu.my (F.H.F.J.); helmy@ukm.edu.my (M.H.M.); 3 Department of Pharmacology, Faculty of Medicine, Universiti Kebangsaan Malaysia, 56000 Kuala Lumpur, Malaysia; chinkokyong@ppukm.ukm.edu.my; 4 Centre of Diagnostic Science and Applied Health, Faculty of Health Sciences, Universiti Kebangsaan Malaysia, 43600 Bangi, Malaysia

**Keywords:** female reproductive system, honey, ovariectomy, hormone, surgical menopause, phytochemical properties of honey, aging, digestive syndromes

## Abstract

Phytochemical contents of honey are presumed to be beneficial to the female reproductive system (FRS). However, the biological effects of honey supplementation (HS) in vivo on the FRS remain unclear. This review aims to investigate the current literature on the effects of HS on the FRS, particularly on the sex hormone profile and reproductive organs (uterus and vagina). A systematic literature search using Scopus, MEDLINE via Ovid and Cochrane Library databases was conducted. Records were screened and identified for preclinical and clinical studies addressing the effects of HS on the FRS. Data on populations, interventions, outcomes and methodological quality were extracted. Studies were synthesised using tables and written summaries. Of the 198 identified records, six fulfilled the inclusion criteria. All six records were used for data extraction: two experimental studies using rats as the model organism and four human clinical studies of honey on female reproductive health. HS elevated the progesterone levels, restrained body weight increase, prevented uterine and vaginal atrophies in ovariectomised rats, attenuated symptoms of candidiasis and improved oxidative status in patients. Current evidence shows that short-term HS following surgical or physiological menopause exerts an oestrogenic, antioxidant and anti-inflammatory effect on the FRS. However, insufficient long-term studies preclude any definitive conclusions.

## 1. Introduction

The use of honey for general health found its beginning approximately 8000 years ago [1]. The use of honey to resolve numerous indications associated with different organ systems, ranging from irritating cough, respiratory tract infection, cataract and ocular diseases, fever and insomnia, has been documented in ancient texts [2,3].

Modern scientific evidence further consolidates the medical benefits of honey. For instance, with regard to the circulatory system, honey is a potent source of trace elements, such as iron (Fe), copper (Cu) and manganese (Mn), necessary for haemoglobin synthesis. Honey supplementation (HS) reduces the severity of anaemia [4,5] and angina resulting from ischemia [6,7]. Honey also enhances the integumentary system by accelerating wound healing and preventing sepsis and alopecia [8].

Honey has been used as a female contraceptive. In ancient Mesopotamia and Egypt, honey mixed with acacia leaves and lint was placed in the vagina to inhibit fertilisation by occluding sperm entry to the reproductive tract [2]. The Kahun Gynaecological papyrus detailed the use of honey and sodium carbonate in a mixture to be applied to the entrance of the cervix. In India, honey was used as a pessary of soluble substance with contraceptive properties [3]. However, recent findings have indicated that honey is a biphasic phytoestrogen exhibiting an anti-oestrogenic effect at lower concentrations and an oestrogenic effect at higher concentrations via binding to the oestrogen receptors [9]. This property of honey can be used to improve the female reproductive system in addition to its contraceptive function.

The medicinal properties of honey are attributed to its complex composition, ranging from trace elements, such as minerals, enzymes and antioxidants such as flavonoids and polyphenols, to carbohydrates [10,11]. In addition to the minerals aforementioned, other minerals commonly detected in honey are cadmium (Cd), lead (Pb), chromium (Cr), nickel (Ni) and zinc (Zn) [12]. Myriad enzymes are also commonly detected in honey, such as diastase (amylase), invertase and glucose oxidase, which contribute to the therapeutic effect of honey [5,8,13]. Antioxidant compounds such as flavonoids (quercetin and kaempferol) and phenolic acids (vanillic acid, gallic acid and syringic acid) contribute to the anti-oxidative property of honey [14] by quenching of reactive oxygen species (ROS), thereby preventing free radical formation [15,16]. Oxidative stress (OS) is neutralised following HS as it increases the glutathione peroxidase (GPx), reduces glutathione (GSH), superoxide dismutase (SOD) and nitric oxide (NO) levels [17]. In addition, many studies have reported the anti-inflammatory, antimicrobial and immunomodulatory properties of honey [18,19].

Evidence on the effect of honey on the reproductive system is sparse, apart from its ancient application as a female contraceptive agent [2]. Other reported applications of honey in gynaecological problems include its anti-fungal properties in genital tract infection [20], pain relief for primary dysmenorrhoea [21] and collagen-promoting action in the prevention of premature rupture of foetal membranes [22]. This review aims to assess the effects of HS on the female hormone profile and reproductive organs (uterus and vagina) to consolidate the current understanding of this field. This contribution to the body of knowledge aspires to enlighten the potential for honey to be developed as an alternative treatment for female reproductive disorders.

## 2. Evidence Acquisition

### 2.1. Data Sources and Search Strategy

A computerised literature search was conducted to identify the available evidence on the effect of HS on the reproductive organs and hormonal profile of women. The search was performed using MEDLINE via Ovid (1946 to February 2020), Scopus and Cochrane with the following search string: honey AND uterus OR uterine OR ovary OR vagina OR progesterone OR oestrogen OR “follicle-stimulating hormone” OR FSH OR “luteinizing hormone” OR LH. Duplicated documents were removed upon merging the search results of individual databases. The PRISMA checklist for systematic literature review was included as Supplementary Table S1. This study is an analysis of currently available data. Hence, no ethical approval was required.

### 2.2. Data Study Exclusion and Inclusion Criteria

The articles were reviewed for eligibility by two reviewers independently (N.H.I. and F.H.F.J.). Disagreements were resolved through consensus by the inclusion of a third reviewer (S.F.I.). The articles were considered on the basis of the following inclusion criteria: full-length original articles published in English, using an animal model (rat or mouse) or human subjects (menopausal/ovariectomised/non-menopausal/non-ovariectomised) and involving a study group solely receiving HS. Studies using other bee products, such as royal jelly, propolis and chrysin, were excluded. A clinical trial published as conference submission, sourced from the Cochrane Library, was included because of the limited human studies.

The protocols of the clinical trial were accessible from the Iranian Registry of Clinical trials: http://www.irct.ir (Clinical trial IRCT2013071013933N1; 14 July 2013 and Clinical trial IRCT201604144317N9; 28 June 2016). The two reviewers (N.H.I. and F.H.F.J.) extracted data on the study groups, interventions, comparators, outcomes and clinical/experimental methodological design independently.

### 2.3. Data Extraction and Management

The article selection was a two-step process. In step 1, articles were screened on the basis of titles and abstracts. In step 2, the full texts of articles filtered from step 1 were screened for eligibility. Differences in opinions on the eligibility of an article were resolved by consensus with two additional reviewers (K.O. and S.F.I.). Data extracted from the articles included sample size, treatment, route, dose and duration of HS, source and type of honey, age of animals in animal study or characteristics of human subjects involved in the clinical trial, assays used to assess hormonal profiles and changes in hormonal levels after honey consumption. Primary outcomes assessed were the effect on female reproductive hormones and tissues (uterine or vaginal tissue-associated observation). Corresponding authors were contacted when data on outcomes of clinical trials were not available. All human clinical trials were based on a double-blinded clinical trial or randomised controlled clinical trial approach, using standard treatment for a condition as control. Studies involving animals reported control treatments as baseline control without HS or OVX surgery control without HS.

## 3. Principal Findings

**Figure 1:**
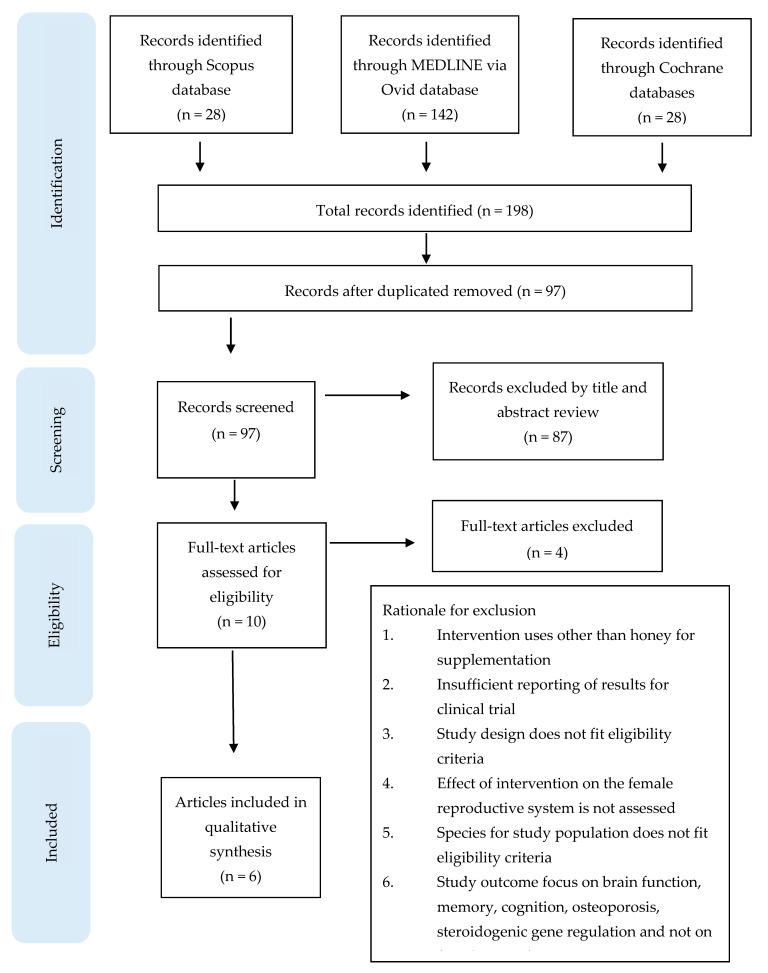
Flow chart of the article selection process from Scopus, MEDLINE via Ovid and Cochrane databases.

**Figure 2:**
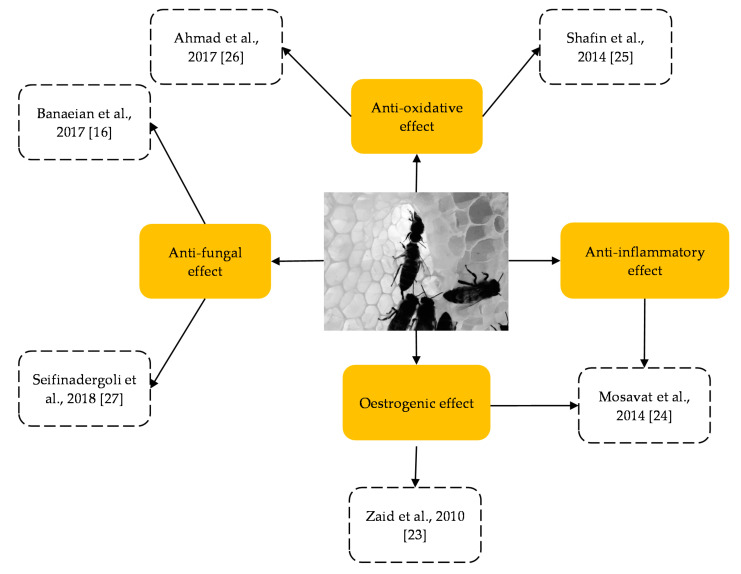
Categories of biological activity resulting from honey supplementation on the female reproductive system. Literature from the systematic review revealed that the six studies on the effect of honey supple mentation were clustered into four main biological effects.

The literature search generated 198 records, of which 101 duplicates were removed, resulting in 97 unique records. The titles and abstracts of the 97 articles were reviewed for eligibility, and only ten articles were eligible for consideration. Subsequent screening of the full texts resulted in four records being removed attributable to the rationale listed in Figure 1. Six records conformed to the inclusion criteria, which were included in this review. The intervention characteristics of all studies are described in Table 1. Table 2 and Table 3 list the findings from studies discussing the effects of HS in rodents and human subjects, respectively, on the female reproductive system. The review summarises four major biological activities of honey on the female reproductive system: anti-inflammatory, proliferation (oestrogenic effect), antioxidant and anti-fungal effects (Figure 2). The female reproductive organs studied were the uterus, vagina, ovary and female hormones in body fluids (blood).

In this review, four studies [23,24,25,26] utilised Tualang honey, identified as multi-floral honey collected by *Apis dorsata* bee species in the north-western region of Peninsular Malaysia (419 m above sea level). Two other studies used Iranian honey. Honey collected from the Ardebil City around Sabalan Mountain in north-western Iran (1315 m above sea level) was utilised in the study by [27]. A study by [16] used honey from the Chaharmahal and Bakhtiar Region of south-western Iran (2072 m above sea level). However, the floral classification of the Iranian honey (either monofloral or multi-floral) was not indicated. All information is summarised in Table 1.

The literature showed that HS could prevent atrophy of the uterus and associated tissues resulting from ovariectomy [23] and jumping exercise in animal models [24]. The effects of HS on reproductive hormones were heterogeneous. It lowered the level of oestradiol and progesterone but increased the level of testosterone in ovariectomised rats [23]. On the contrary, it increased the level of oestradiol but did not alter the progesterone level in rats following strenuous exercise [24] (Table 2). The unaffected level of progesterone in the study [24] is thought to be due to the stress-attenuating effects of HS which mitigated oxidative imbalance during strenuous exercise.

HS also exhibited anti-fungal effects on vulvovaginal candidiasis (VVC) infections in humans. Honey in cream and gel formulation decreased discharge, inflammation and itching amongst patients with the infection [16,27]. It also reduced the rate of recurrent infections amongst women [16]. The effects of HS were found to be comparable with clotrimazole (a standard treatment) in reducing the symptoms related to VVC [27].

Other reports indicated that HS improved the oxidative status of menopausal subjects and female athletes (Table 3) by enhancing the antioxidant enzymes [25] and reducing lipid peroxidation products [25,26].

The female reproductive system was governed by peptide (gonadotropins: follicle-stimulating hormone, FSH and luteinizing hormone, LH) and steroid (oestrogen, progesterone and testosterone) hormones. The peptide hormones, FSH and LH, were hydrophilic, which elicited cellular response via binding with G-protein-coupled receptors (GPCRs) and activation of the cyclic AMP (cAMP) second-messenger pathway and concurrent pathways, such as inositol trisphosphate (IP3) and diacylglycerol (DAG) [28]. The binding of either FSH or LH with GPCRs on the membrane surface caused shuffling of the α-subunit of G-protein and activation of adenyl cyclase, thereby causing cAMP to activate kinase A to phosphorylate designated proteins and elicit various cellular responses. Each pathway activated a certain cellular response in the uterus and ovary, such as relaxation or contraction of uterine tract motility [29], gamete production and secretion of sex hormones [30]. By contrast, the lipophilic steroid hormones stimulated genes within the target cell by binding with intracellular receptors, thereby promoting the synthesis of new proteins to elicit cellular responses [30]. Cellular responses within the female reproductive system, such as ovulation, menstruation, implantation and parturition, were characterised by inflammatory responses, which might include the activation of the chemokine, cytokine and mitogen-activated protein kinase/nuclear factor κB (MAPK/NFκB) pathway [29]. The cellular responses governed by the above-mentioned pathways are discussed in the following sections.

The direct oestrogenic effects of honey on female reproductive organs have been shown in several studies. In the reports by Zaid et al. [23], 3-month-old Sprague Dawley rats supplemented with Tualang honey at different concentrations showed that low-dose HS improved epithelial thickness, vaginal and uterus weights and antioxidant capacity. They concluded that 0.2 g/kg body weight (bwt) and 1.0 g/kg bwt of honey for 2 weeks were the optimal regimes to achieve these effects. This result indicated that honey possessed oestrogenic properties that prevented atrophy of uterine and vaginal epithelia resulting from oestrogen deficiency in instances of surgical menopause induced by ovariectomy (OVX). Flavonoids and phenolic acids were responsible for the oestrogenic effect of HS [24], which contributed to relieving the symptoms associated with menopause. The study also reported that rats, which were subjected to intense physical activity and given HS, retained their uterine and vaginal weights. Furthermore, HS increased the progesterone levels and, to a lesser extent, the oestrogen level in the supplemented rats [24]. On the contrary, Zaid et al. [23] reported a decline in the oestrogen levels of rats following HS compared with the OVX and sham-surgery groups. The level of oestrogen reduced in a dose-dependent manner, which was the lowest in the group receiving the highest concentration of HS. The reason for this change was unknown.

**Table 1:** Intervention characteristics of honey supplementation.

Type of HS (No. of HS Group)	Dosage of HS	Route of HS	Duration of HS	Target Female Reproductive Organ/Hormone of Study	References
Tualang honey (3)	0.2 g/kg bwt, 1.0 g/kg bwt and 2.0g/kg bwt	Oral route	2 weeks	Uterus (relative weight and thickness) and vagina (relative weight and thickness); oestrogen and progesterone levels	[23]
Tualang honey (1)	1 g/kg bwt/day	Oral route	8 weeks	Uterus (relative weight); oestrogen and progesterone levels	[24]
Tualang honey (1)	20 g/day	Oral route	16 weeks	None. The study measured blood GSH (reduced glutathione) to GSSG (oxidised glutathione) ratio, CAT, SOD, GPx, 4-HNE and total protein	[25]
Tualang honey (2)	0.75 g/kg bwt and 1.5 g/kg bwt	Oral route	5 h	None. The study measured TPC, antioxidant stress activity and oxidative stress markers (MDA and ROS)	[26]
^a^ Chaharmahal and Bakhtiari region, Iran honey (1)	5 g of 70% honey cream (with applicator)	Topical route	7 nights	Reproductive tract. The study measured symptoms of VVC: inflammation, discharge, irritation and the level of satisfaction of treatment	[16]
^b^ Sabalan Mountain, Iran honey (1)	5 g of 50% honey gel (with applicator)	Topical route	8 nights	Reproductive tract. The study measured symptoms of VVC: vaginal discharge, itching, dyspareunia, burning, urinary incontinence and culture of vaginal sample to confirm the reduction in *Candida albicans* colony number infection	[27]

The table provides a summary of intervention used in each study to test the effect of honey supplementation on female reproductive organs or hormones. The interventions are described on the basis of the route of honey supplementation, the duration of honey supplementation given and the target female reproductive organ or female hormone, for which the effect was most observed. bwt, body weight; HS, honey supplementation; VVC, vulvovaginal candidiasis; GSH, glutathione (reduced form); GSSG, glutathione (oxidised form); CAT, catalase; SOD, superoxide dismutase; GPx, glutathione peroxidase; 4-HNE, 4-hydroxynonenal; TPC, total phenolic content; FRAP, ferric reducing antioxidant power; MDA malondialdehyde; ROS, reactive oxygen species. ^a^ Honey was dissolved in neutral cream; ^b^ honey was dissolved in neutral gel.

**Table 2:** Effect of honey supplementation on the female reproductive system in rodents.

Sample/Subject	Sample Size (n) Per HS Group; Total Number of Groups Given HS in Study; Number of Control Individuals	Age of Sample/Subject	Effect of HS on the Female Reproductive System	Study Sample/Subject is Menopause, OVX or Normal Rats	Biological Effect of Honey on the Female Reproductive System	References
Sprague Dawley rats	7/group; 3 groups; 14	12 week old	HS prevented the ovariectomised rats from uterine atrophy and vaginal epithelium atrophy. The oestradiol and progesterone levels were significantly decreased in the HS group. The testosterone level was increased in the low-HS group (0.2 g/kg bwt). Effects on other than the female reproductive system: promoted bone density and suppressed body weight increase	OVX rats	Oestrogenic effect	[23]
Sprague Dawley rats	12/group; 3 groups; 24	9 week old	HS effectively maintained relative uterine weight and attenuated the adverse effects of jumping exercise on the female reproductive hormone (the increase in the oestradiol levels was not significant, whereas the increase in the progesterone levels was significant) and stress hormones (cortisol decreased but not significant). No effect on relative ovary weight.	Non-menopausal, Non-OVX (Normal rats)	Oestrogenic effect and anti-inflammatory	[24]

Studies on rodents (Sprague Dawley rats) included for the systematic review were primarily experimental studies with a limited representation of the age group. The related biological effect following honey supplementation was identified. HS, honey supplementation; OVX, ovariectomised.

**Table 3:** Effect of honey supplementation on the female reproductive system in human subjects.

Sample/Subject	Sample Size (n) Per HS Group; Total Number of Groups Given HS in Study; Total Sample Size (N) for Study	Age of Sample/Subject	Effect of HS on the Female Reproductive System	Study Sample/Subject is Menopause, OVX or Non-Indicated	Biological Effect of Honey on the Female Reproductive System	References
Human (postmenopausal)	39/group; 1 group; 78	45–60 years old	HS resulted in significant elevation of CAT and GPx activity and reduction in the 4-HNE levels. SOD activity and GSH/GSSG ratio were insignificant.	Menopause subjects	Anti-oxidant	[25]
Human (athletes)	10/group; 1 group; 20	18–25 years old	HS significantly lowered the MDA levels. The changes in the TPC, FRAP and ROS levels were insignificant.	Non-indicated	Anti-oxidant	[26]
Human (VVC patient)	44/group; 1 group; 80	21–59 years old	HS (in cream formulation) showed a significant decrease in discharge, inflammation and itching. However, the magnitude was less than control (clotrimazole cream). HS showed a lower rate of recurrence (in women with more frequent VVC history) during follow-up.	Non-indicated	Anti-fungal	[16]
Human (VVC patient)	53/group; 1 group; 106	18–45 years old	HS (in gel formulation) has significantly reduced the problems associated with VVC (symptoms of itching, burning, dyspareunia, urinary problems and vaginal discharge), and culture plate showed no colonies of *Candida albicans*. Results of HS was comparable or having nearly equal effect to a standard treatment; clotrimazole cream group.	Non-indicated	Anti-fungal	[27]

Studies on human subjects included for the systematic review were primarily clinical studies, with a comprehensive representation of the age group. The related biological effect following honey supplementation was identified. HS, honey supplementation; VVC, vulvovaginal candidiasis; GSH, glutathione (reduced form); GSSG, glutathione (oxidised form); CAT, catalase; SOD, superoxide dismutase; GPx, glutathione peroxidase; 4-HNE, 4-hydroxynonenal; TPC, total phenolic content; FRAP, ferric reducing antioxidant power; MDA, malondialdehyde; ROS, reactive oxygen species; OVX, ovariectomised.

## 4. Discussion

### 4.1. Role of Honey in Suppressing Oestrogen Deficiency-Induced OS

In the female reproductive system, OS and the formation of ROS occur in the ovary, uterus and placenta. OS affects several key functions, including ovarian steroid genesis, luteal maintenance during gestation, modulation of ovarian germ cell and physiology of menstrual cycle. Nevertheless, it has a dual effect, whereby ROS may promote or hinder the normal physiology of the female reproductive system. ROS is important to the female reproductive system as it promotes the development of follicles, encourages ovulation and suppresses the effects of apoptosis, endometrial shedding, luteum dissolution and synthesis of female hormones, such as progesterone and oestrogen [31]. However, excessive ROS leads to a disruption in the balance of free-radical-to-antioxidant ratio and promotes OS, leading to impairment of female fertility [32]. Antioxidants in the female reproductive system may act either via the primary or secondary defence mechanisms that prevent the dismutation of oxygen radicals or neutralise lipid peroxidation to restore oxidative balance [33]. The decline in the oestrogen level seems to be the major factor in elevated OS in women as it marks an increase in endogenous ROS. HS impedes free radical formation because it contains numerous components with free radical scavenging activities. The antioxidant potential of honey was evaluated by Shafin et al. [25] and Ahmad et al. [26]. Both studies reported that supplementation of Tualang honey at lower doses enhanced the physiological antioxidant capacity. A study by Shafin et al. [25] reported 20 g of Tualang honey/day and [26] recorded 0.75 g of Tualang honey/kg bwt as the optimal dosages with the greatest antioxidant effects. Shafin et al. [25] evaluated the anti-oxidative effects of honey in menopause subjects (45–60 years old). By contrast, the subjects in the study by Ahmad et al. [26] were young athletes (18–25 years old). In both studies, HS could boost the antioxidant status of both cohorts despite their differences with regard to age and body physiology. HS was able to withstand the increased OS following intense physical activity [26]. In addition, lipid peroxidation markers were reduced, which further consolidated the findings on the antioxidant capacity of HS. Furthermore, an indirect correlation was found between the time elapsed after HS and its antioxidant effects. Ahmad et al. [26] revealed that HS increased the antioxidant capacity and reduced the OS markers until 2 h post-administration.

Mosavat et al. [24] showed that OS was increased following strenuous exercise, resulting in functional disruption of the human female reproductive system. The disruption in the hypothalamic–pituitary–ovary (HPO) axis affects the pulsatility of GnRH, which subsequently induces amenorrhoea. The HPO axis regulates reproductive development and function achieved by pulsatile stimulation upon the release of GnRH [33]. However, the HPO axis is vulnerable to ROS, and GnRH pulsatility can be compromised with OS, for instance, during intense physical activity.

Intense physical activity increases the concentration of serum cortisol and reduces the level of available energy, thereby leading to an accumulation of ROS and physical and emotional stress. It also increases the risk of infections and inflammation, which in turn depletes oestrogen synthesis and compromises the endogenous antioxidant system [24,26]. The antioxidant capacity of flavonoids and phenolic acids in honey, such as kaempferol and quercetin, can attenuate the detrimental effects of strenuous exercise and restore hormonal levels [24].

### 4.2. Role of Honey in Suppressing OS-Induced Inflammation Stress

The elevated levels of free radicals have been found to activate the nuclear factor κB (NFκB) pathway responsible for eliciting inflammation. The activation of the NFκB pathway increases the prostaglandin F2-alpha (PGF2α) level, which exerts hormone-like activity by promoting luteum dissolution [33]. In the uterus, the activation of the NFκB pathway causes endometrial shedding because of reduced steroid genesis (progesterone and oestrogen) in the late luteal phase and endothelial cell dysfunction, resulting in preeclampsia and endometriosis. Furthermore, in preeclampsia cases, placental NFκB is activated at levels of up to tenfold [34], leading to fertility with life-threatening complications.

In addition, oestrogen deficiency activates the MAPK/NFκB pathway [25]. Notably, oestrogen exhibits antioxidant properties because of its interaction with oestrogen receptors, and a change in its homeostasis leads to the accumulation of ROS and elevates the oxidative levels. Subsequently, the elevated oxidative level causes stimulation of kinase from the Src family and activates the MAPK signalling pathway. MAPK also activates serine residues of NFκB subunits via phosphorylation. This activation of the MAPK/NFκB pathway and up-regulation of p53 target genes corresponding to elevated OS negatively affect the physiology of the female reproductive system [35].

Mosavat et al. [24] showed that HS exerted anti-inflammatory effects in rats undergoing jumping exercise by quenching of ROS resulting from the physical activity. Intense physical activities also activate the hypothalamic–pituitary–adrenal (HPA) axis, thereby stimulating the production of serum cortisol. Elevated serum cortisol is implicated in suppressing the HPO axis, causing HPO-associated amenorrhoea [36]. Honey supplementation suppresses the further increase in cortisol levels induced by intense physical activities [24].

### 4.3. Role of Honey as an Anti-Fungal Agent

Honey is effective against fungal and bacterial growth because of its physicochemical attributes, such as hypertonicity (due to high sugar content), high acidity and the presence of phytochemicals (flavonoids and alkaloids such as propolis, chrysin, pinobanksin, galangin, quercetin, luteolin and kaempferol) [37,38]. Glucose oxidase in honey also produces hydrogen peroxide as a by-product of glucose oxidation, which exerts a fungicidal effect [39]. The hygroscopic and hyperosmolar properties of honey contribute to its anti-fungal effects by increasing the osmolarity of the immediate environment to which honey is being supplemented [40].

Honey has been shown to exert remarkable anti-fungal effects against *Candida* sp. in VVC, which alters the reproductive capacity of the female reproductive system by increasing OS. Innate immune cells produce toxic ROS as a form of the fungicidal mechanism via the assembly of NADPH oxidase complex stimulated by cytokines released by the cells [41]. However, *Candida* sp. counteracts this body defence mechanism by expressing antioxidant enzymes on its surface, which spare the fungi from the effects of ROS and allow the infection to worsen. The elevation of endogenous ROS poses a detrimental effect on the reproductive function instead of controlling the infection. VVC infection leads to inflammation of the mucosal membrane of the reproductive tract. VVC is common and presents a major risk to patients as it may induce irreversible damage to the reproductive organs, such as dyspareunia (pain ensuing from sexual intercourse) and climacteric symptoms (menstrual bleeding disorders, vaginal dryness and metabolic syndrome). These symptoms require an excessive cost of treatment to overcome the recurring infection amongst the patients [16,27] and other emerging medical symptoms. If the infection is untreated, then irreversible infertility caused by loss of function is possible. Banaeian et al. [16] and Seifinadergoli et al. [27] reported the anti-fungal properties of honey used either as a cream or gel formulation. The clinical studies on VVC infection showed better outcomes following treatment with 50% honey gel for eight nights [27] compared with 70% honey cream for seven nights [16]. In general, both treatments could relieve the symptoms of VVC compared with clotrimazole control. However, 50% honey gel showed comparable results against control, with no recurrent fungal infection identified by colony counting on culture plates from vaginal swab samples. The honey gel given was water-based, allowing the honey to dissolve better in the mixture and enabling faster absorption by the mucosal membrane, thereby improving the anti-fungal effect compared with the honey cream. The oestrogenic, antibacterial and anti-fungal effect of honey inhibits pain caused by dyspareunia symptoms, including vaginal dryness, vaginal infection or vaginitis [8,16,17,18,21].

Honey contains oligosaccharides that can be utilised by saccharolytic fermenters to yield beneficial metabolites that promote the probiotic effect [42]. High oligosaccharide content in honey facilitates healthy gut microbiota (particularly bifidobacteria). Bifidobacteria is a probiotic that prevents the growth of harmful pathogens, which may cause leaky gut, when combined with endogenous phenols and hydrogen peroxide [43].

Bifidobacteria are Gram-positive, anaerobic bacteria ubiquitous in the mammalian gastrointestinal tract, vagina and oral cavity. The *Bacillus* group [44] is the dominant species of bacteria isolated from honey. Bifidobacteria are beneficial antimicrobials to balance the vaginal microflora as they produce biosurfactants that repel pathogenic bacteria, which are dependable probiotics [45]. In a healthy vagina microenvironment, dominancy in *Lactobacillus* sp. is observed [46]. Regulation of gastrointestinal and vaginal flora with probiotics may prevent genitourinary infections. Probiotics used must be resistant to gastric and bile acids to reach the intestinal system and produce beneficial effects. Some *Lactobacillus* sp. produce hydrogen peroxide and biosurfactants that acidify vaginal mucosa and prevent urogynecologic infections, thereby exhibiting strong inhibitory effects towards *Escherichia coli*.

Honey produces endogenous hydrogen peroxide by hydrolysis of glucose using glucose oxidase. It has gained interest as an alternative therapy or multi-drug treatment and prophylaxis of urogenital infections. Urogenital infections are characterised by the presence of uropathogenic *E. coli*. *E. coli* was reported to colonise the uroepithelia in the vagina and ascend to the uterine horns resulting in asymptomatic bacteriuria in recurring urinary tract infections [47]. Good probiotics must bind to uroepithelial cells and inhibit pathogenic growth and biosurfactant secretions. Frequency of sexual intercourse multiplies the risks of recurring urogenital infections.

Earlier studies of HS on gynaecological problems focused on the applications of HS as (i) an anti-fungal agent for genital tract infection [20,27,28,43], (ii) pain relief for primary dysmenorrhoea [21] and (iii) collagen-promoting actions of honey in the prevention of premature rupture of foetal membranes [22]. Although these studies were conducted on the female reproductive system, no reports were made on the effects of HS on the female hormonal levels, menstrual cycle and symptoms of menopause.

### 4.4. Is Honey Suitable for Patients with Hyperglycaemia?

A point of concern with HS is the high sugar content of this functional food. According to previous reports, honey is beneficial as a dietary supplement to reduce blood glucose levels [48]. Nevertheless, it is ineffective in reducing glycohaemoglobin (HbA1c) concentrations [49]. HS improves glycaemic control, exerts a hypoglycaemic effect in normal and diabetes-induced rats and further enhances the effects of anti-diabetic drugs when taken simultaneously [50,51]. Similar effects are observed in human subjects with diabetes [49,52,53,54,55,56,57,58,59], and the therapeutic properties of HS are contributed by the fructose-to-glucose ratio [48]. Fructose will undergo the first-pass removal in the liver during carbohydrate metabolism; thus, it is responsible for the hypoglycaemic effect [60,61]. Notably, fructose metabolism is independent of insulin and is the key to activating glucokinase for glucose metabolism via the conversion of glucose to glucose-6-phosphate to produce hypoglycaemic effects [62]. However, short-term application of honey can result in increased blood glucose levels [49]. Moreover, antioxidant compounds in honey exert an anti-obesity effect, which is favourable for diabetics as it attenuates insulin resistance. The anti-obesity effect of honey in adipocytes is demonstrated by coumaric and caffeic acids via arresting adipocytes in the G1 phase of cell cycle and inhibiting fatty acid synthase [63].

Honey’s anti-lipidaemic and anti-inflammatory properties exerted by phenolic antioxidants regulate and maintain body weight via induction of thermogenesis [43]. Consumption of natural honey in overweight and obese subjects was shown to suppress body weight increase and reduce cardiovascular risk factors, thereby conferring cardioprotective effects in overweight and obese subjects [64]. Oestrogen receptors additionally mediated the effects on hepatic expression of apoproteins, which mediate serum lipoproteins. Mendhelson and Karas [65] reported that oestrogen therapy (ET) in postmenopausal women has been shown to reduce serum total cholesterol and low-density lipoprotein (LDL) concentrations, increase serum high-density lipoprotein (HDL) and triglycerides and predominantly reduces serum lipoprotein concentration. Orally administered ET showed favourable results over transdermal administration.

Notably, these properties may only be reproducible via the standardisation of honey for product development. Nordin et al. [66] expressed the same concerns with regard to the use of stingless bee honey. Table 4 summarises the active compounds and physico-chemical attributes of honey with oestrogenic, anti-inflammatory, antioxidative and anti-fungal activities.

## 5. Conclusions

Honey is a widely accepted natural food, which exerts multiple functions on the female reproductive system. Thus, it can be promoted as a functional food to menopausal women as an alternative therapy for female reproductive system disorders. The current review summarises evidence on the oestrogenic, anti-oxidative and anti-fungal effects of honey. Clinical studies on the effects of HS on the female human reproductive system require extensive validation through further studies. Some challenges in developing honey as a functional ingredient to prevent or treat gynaecological problems include standardising of the constituents and dose of natural honey, the proliferation of artificial honey in the market and conservation issues of honeybees. Its safety amongst low-fertility and menopausal female patients with co-morbidities such as diabetes also needs further validation. These issues must be considered when developing honey as a product to promote women’s health.

## Figures and Tables

**Table 4:** Summary of active compounds and physico-chemical attributes of honey with oestrogenic, anti-inflammatory, antioxidative and anti-fungal activities.

Biological Activity	Active Compounds (AC) and Physico-Chemical Attributes (PCA) of Honey	Biological Effect on Female Reproductive Disorder
Oestrogenic	**AC**: Flavonoids and phenolic acids [24]	alleviate symptoms associated with menopause [24]
Anti-inflammatory and antioxidant	**AC**: Phenols, flavonoids, ascorbic acid, α-tocopherol, carotenoid compounds, enzymes, Maillard reaction products between reducing sugars and amino acids [67]	increases the vitamin C levels, trace element absorption and antioxidant capacity in the plasma and brain [43] increases brain-derived neurotrophins that improve spatial memory, anxiety, behaviours and symptoms associated with depression and stress response by lowering the blood cortisol levels [43]
	**AC**: Choline and acetylcholine [68]	protection against memory decline caused by stress and ageing by reducing the brain OS levels and increasing the expression of brain-derived neurotrophic factor (BDNF) [68] Honey treatment reduces the corticosterone, adrenocorticotropic hormone, MDA and protein carbonyl levels and improves memory performance, SOD and BDNF concentration [68] treatment of neurodegenerative conditions via modulation of oestrogen effects, oestrogen-metabolising enzyme expression and oestrogen receptor expression [35].
Anti-fungal	**PCA**: hypertonicity (due to high sugar content), high acidity [37,38], hygroscopicity and hyperosmolarity properties [40] **AC**: propolis, chrysin, pinobanksin, galangin, quercetin, luteolin and kaempferol [51,52], glucose oxidase [53], oligosaccharides [56]	anti-fungal effect by increasing the osmolarity of the immediate environment to which honey is being supplemented [40] fungicidal effect from hydrogen peroxide produced as a by-product of glucose oxidation [39] probiotic effect from oligosaccharides that are utilised by saccharolytic fermenters to yield beneficial metabolites [42] high-oligosaccharide content in honey facilitates healthy gut microbiota (particularly bifidobacteria) [43]

## Supplementary Materials

The following are available online, Table S1: PRISMA Checklist.
